# Polyploidy can Confer Superiority to West African *Acacia senegal* (L.) Willd. Trees

**DOI:** 10.3389/fpls.2016.00821

**Published:** 2016-06-14

**Authors:** Adja M. Diallo, Lene R. Nielsen, Erik D. Kjær, Karen K. Petersen, Anders Ræbild

**Affiliations:** ^1^Department of Geosciences and Natural Resource Management University of Copenhagen, Denmark; ^2^Centre National de Recherches Forestières/Institut Sénégalais de Recherches AgricolesDakar, Senegal; ^3^Department of Food Science, Aarhus UniversityAarslev, Denmark

**Keywords:** adaptation, arid zone trees, drought stress, flow cytometry, microsatellite markers, morphological differentiation, *Senegalia senegal*

## Abstract

Polyploidy is a common phenomenon in the evolution of angiosperms. It has been suggested that polyploids manage harsh environments better than their diploid relatives but empirical data supporting this hypothesis are scarce, especially for trees. Using microsatellite markers and flow cytometry, we examine the frequency of polyploids and diploids in a progeny trial testing four different populations of *Acacia senegal*, a species native to sub-Saharan regions of Africa. We compare growth between cytotypes and test whether polyploid seedlings grow better than diploids. Our results show that polyploids coexist with diploids in highly variable proportions among populations in Senegal. *Acacia senegal* genotypes were predominantly diploid and tetraploid, but triploid, pentaploid, hexaploid, and octaploid forms were also found. We find that polyploids show faster growth than diploids under our test conditions: in an 18 years old field trial, polyploid superiority was estimated to be 17% in trunk diameter and 9% in height while in a growth chamber experiment, polyploids grew 28% taller, but only after being exposed to drought stress. The results suggest that polyploid *A. senegal* can have an adaptive advantage in some regions of Africa.

## Introduction

Polyploidy, the achievement of more than two sets of chromosomes through gametic non-reduction and to a lesser degree somatic doubling has important ecological and evolutionary consequences for speciation (Madlung, 2013). In nature, polyploidy arises via intraspecific genome doubling (autopolyploidy) or merging of genomes of distinct species through hybridization and chromosome doubling (allopolyploidy) (Stebbins, 1950). Most angiosperms are believed to have undergone one or more polyploidization events (Soltis and Soltis, 1995). It has been estimated that polyploids form at the frequency of approximately 1 per 100,000 individuals (Ramsey and Schemske, 1998; Levin, 2002) and that 2–4% of all speciation events involve polyploidization (Otto and Whitton, 2000). The high level of polyploidization in the evolutionary history of flowering plants suggests that polyploidy plays an important role in adaptive evolution of plants in natural populations (Van de Peer et al., 2009). Successful polyploidization is generally accompanied by morphological, phenological, physiological, and ecological changes in plants (Levin, 2002), and may produce individuals that can tolerate fluctuating environments (Soltis et al., 2004; Prentis et al., 2008), make use of new niches or by other means become more successful than their progenitor species (Leitch and Leitch, 2008).

In theory, local co-occurrence of intraspecific cytotypes is evolutionary unstable, driving the minority cytotype toward extinction unless cytotypes have different ecological preferences or strong prezygotic barriers are present between ploidy levels (Husband, 2000; Kennedy et al., 2006). Nevertheless, co-distribution of individuals belonging to different ploidy levels in heteroploid species (species with different levels of ploidy) is not uncommon and has been reported in, e.g., *Solidago altissima*, *Ranunculus parnassifolius*, and *Centaurea phrygia*, *C. stoebe* (Halverson et al., 2008; Cires et al., 2010; Koutecky et al., 2012). With the recent introduction of flow cytometry is it now possible to explore the cytotype dynamics in species with mixed ploidy levels in terms of cytotype distribution, hybridization, and segregation.

Morphological changes in polyploids include a general increase of cell size with increased levels of ploidy (e.g., Kudo and Kimura, 2002), sometimes leading to changes in the dimensions of plants, such as larger leaf, flower and fruit sizes compared to diploids (Maherali et al., 2009; Pettigrew et al., 2012). Also micro-morphological changes occur, including larger but more dispersed stomata in polyploids than in diploids (Mishra, 1997; Pettigrew et al., 2012). Such changes are likely to affect plant environment interactions, for example, through modification of gas exchange. It was earlier suggested that polyploids withstand harsh environments like subarctic regions, high elevations and xeric environments better than diploids (Love and Love, 1949) perhaps due to their higher levels of heterozygosity and genetic diversity (Lowry and Lester, 2006). This was supported for example by observations of higher colonization potentials in polyploids, increased frequencies of polyploids from warmer to colder latitudes (Manton, 1934; Hagerup, 1939; Brochmann et al., 2004; Lowry and Lester, 2006) and larger ecological amplitude of polyploids compared to diploids (e.g., Schlaepfer et al., 2010; Liu et al., 2011).

Recent studies have found that polyploid cytotypes in heteroploid species complexes of herbaceous plants have better drought tolerance than their diploid progenitors (*Chamerion angustifolium*, Maherali et al., 2009; *Brachypodium distachyon*, Manzaneda et al., 2012), although Buggs and Pannell (2007) found that diploid *Mercurialis annua* performed better than hexaploids across a range of natural sites and that the cytotypes did not differ in performance under drought stress. Increased drought tolerance may be related to higher resistance toward cavitation in the xylem as discovered in *Atriplex canescens*, a shrub species from the deserts of Southwestern US (Hao et al., 2013). Nevertheless, it is still discussed under which circumstances polyploidy confer higher fitness (Madlung, 2013), and a comparison of many North American diploid and polyploid species showed no significant differences in extent of range or geographical distribution between ploidy levels (Martin and Husband, 2009). Unfortunately very few studies showed the relative performance of diploids and polyploids under controlled conditions (Soltis et al., 2010).

Variation in ploidy level within species is also known from trees (e.g., the *Adansonia digitata*/*A. kilima* complex, Pettigrew et al., 2012; *Betula papyrifera*, Li et al., 1996; *Populus tremula*, Johnsson, 1940; *Acacia mearnsii*, Beck et al., 2003). Because increased cell size is likely to influence hydraulic properties via the influence on conduits (see, e.g., Maherali et al., 2009; Hao et al., 2013), studies on trees with their massive xylem, large size and long potential exposure to climatic extremes are particularly interesting. Yet there are few studies comparing performance of trees with different ploidy levels, and the studies that exist focus on short-term responses of seedlings to stress (Li et al., 1996, 2009). Studies of performance of mature polyploid versus diploid trees are to our knowledge absent.

Recently it was discovered that *Acacia senegal* (L.) Willd. exists in different levels of ploidy. The species grows naturally in the semi-arid sub-Saharan regions of Africa as well as in India and Pakistan and plays an important role in agroforestry systems by providing fuel, fodder for livestock and restoring soil fertility besides producing Gum Arabic. Gum Arabic is a natural exudate collected from branches and stems after tapping during the dry season, and is only produced when the species is grown under dry conditions (Wekesa et al., 2009). The gum provides an important income for rural people. Most of the populations across the distribution area seem to be composed of diploid trees, but tetraploid individuals (2*n* = 4*x* = 52) were discovered in populations from Mali, Sudan and Ethiopia (Assoumane et al., 2013). Due to a limited sample size within each population the ratio between diploid and polyploid individuals was not resolved. Based on chloroplast data, the authors suggest that polyploid *A. senegal* is allopolyploid (Assoumane et al., 2013). The parent species that hybridized with diploid *A. senegal* may have been *A. laeta* reported to be a triploid hybrid (3*x* = 39) between *A. senegal* and *A. mellifera* (Ross, 1979), but the origin and type of polyploidy in *A. senegal* is still not verified.

Investigating a progeny trial with mature *A. senegal*, we discovered that the trees had mixed ploidy levels (Diallo et al., 2015). As the trial was established with the purpose of breeding for increased gum production, the trees were planted in an experimental design and thus represent a unique possibility for studying the long-term performance of di- and polyploid trees. In this paper, we specifically (i) explore the distribution of cytotypes in trees originating from four different locations, and in their corresponding offspring, (ii) compare the growth performance of trees with different ploidy levels, (iii) compare the growth of seedlings with different ploidy level under drought stress, and (iv) compare the morphology of plants with different ploidy levels.

## Materials and Methods

### Study Species

*Acacia senegal* (L.) Willd. is a multipurpose tree that belongs to the family Fabaceae. Recently, it has been suggested to transfer the species to the new genus *Senegalia* (Maslin, 2008). Here we maintain the rule of first priority and thus the name *A. senegal*. The species is described as consisting of the four varieties *senegal*, *kerensis*, *rostrata*, and *leiorhachis* based on differences in inflorescence axis, pod, and tree shape and phenology (Odee et al., 2012). Only *A. senegal* var. *senegal* has been reported in Senegal.

### The Field Trial

The plant material of *A. senegal* used in this study originated from a progeny trial in Senegal established in 1994 in Dahra, Senegal (15° 20’ N and 15° 28’ W). The annual precipitation at the site is approximately 410 mm, and the annual mean temperature is 27°C (climate data estimated from Worldclim based on the 1950–2000 period, see Hijmans et al., 2005). The soil at the site is sandy, and the natural vegetation in the area consists of mainly *A. tortilis* and *Balanites aegyptiaca* (Pontanier et al., 2003).

**FIGURE 1:**
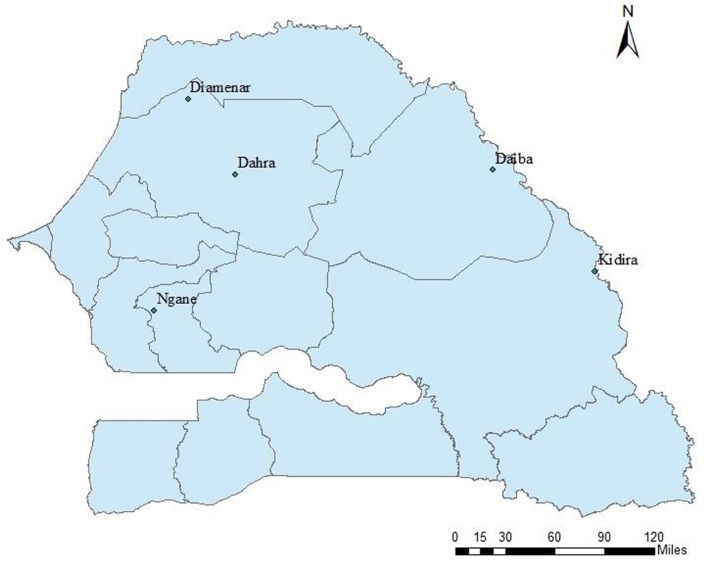
**Location of the progeny trial (Dahra) and the four tested provenances of *Acacia senegal* in Senegal**.

In November and December 1993, seeds were collected from four populations (provenances) representing the natural distribution area of the species in Senegal (**Figure 1**): Ngane, located in the center of Senegal and characterized by saline soils and 620 mm of rainfall; Diamenar, located in a dryer region in the north with 300 mm of rainfall; Daiba and Kidira, located in the north-eastern and south-eastern parts with 430 and 600 mm of rainfall, respectively. At each site seeds were collected from 15 trees considered to have desirable phenotypes based on superior health, trunk diameter, crown diameter, and height. In the natural stands diploids and polyploids are indistinguishable with the naked eye, implying no bias for or against any type during seed collections. Seeds were kept in separate lots for each mother tree, thus giving 60 seedlots, and in 1994 seeds were pretreated in sulfuric acid (98%) for 6 min and sown in polyethylene bags with nursery soil. Two seeds were sown per bag, and in cases where both seeds germinated, one of the seedlings was randomly selected and removed. Seedlings were raised in a nursery and in August 1994 (during the rainy season), 30 healthy seedlings per seedlot were selected and planted at the Dahra site. Prior to plantation, weeding and clearing were undertaken in the site. The trial was established in a randomized complete single tree block design with all seedlots represented by one tree in each block, replicated thirty times (30 blocks). The initial number of plants was thus 4 × 15 × 30 = 1800 trees. Trees were spaced 5 × 5 m from each other.

One year after planting, in August 1995, survival and height of all trees were assessed. Height was measured as vertical height from the ground to the top of the tree. In February 2012, a second assessment was conducted on the 634 surviving trees. The maximum vertical height was determined using a height rod, trunk diameter at 30 cm from the soil surface was assessed using a diameter tape, and crown diameter was estimated as the average of two perpendicular measures of the edge of the crown projected to the ground. In 2012, cambium samples were taken from every tree for assessment of the ploidy level (see later).

### Growth Chamber Experiment

In November 2012, 108 pods were collected from 76 parent trees in the field trial. The parent trees were selected to cover all four sites of origin and – to the extent possible – different levels of ploidy in all sites of origin. One pod was randomly chosen from each tree except for trees from Ngane where 1–6 pods were collected per tree (due to higher frequency of polyploidy in this provenance as described below).

The number of seeds per pod was counted and seed dimensions (length and width) were measured using a Vernier caliper. Seeds were pretreated with sulfuric acid (95–97%) for 10 min to release seed dormancy, kept under sterile conditions in a laminar hood (Thermo Scientific, SAFE 2020, Germany) and germinated in boxes containing sterilized vermiculite and incubated at 29°C and 16 h photoperiod in a growth chamber. The germination rate was registered, and the ploidy level of all seedlings was determined (see below).

To compare the growth between different levels of ploidy, 132 seedlings were transplanted in peat soil (Plugg och Såjord) from Weibulls Horto AB, Sweden. Five hundred grams of soil was filled in 17 plastic boxes each. The seedlings consisted of 83 diploids, 46 tetraploids, and 3 hexaploids. Each box was considered as one block and contained eight seedlings representing different sites of origin (provenance), descendance and ploidy levels. By descendance we here understand all seedlings descending from a single mother tree in the Dahra field trial. Because of the restricted number of polyploid seedlings in some sites of origin (provenances), the design was imbalanced, but each box contained at least three sites of origin and one set of diploids and polyploids from the same site of origin. For example, block no. 1 included four tetraploids and one diploid from the Ngane origin, and one diploid of each of the Daiba, Diamenar, and Kidira origins, while block no. 17 contained one diploid and one tetraploid of the Kidira origin, and three diploids of each of the Daiba and Diamenar origins. Despite the imbalance in the trial, this design allowed us to assess the growth of the different levels of ploidy because there was always at least one pair of dipoid and polyploid seedlings from the same origin in each block. We planted seedlings in boxes to make sure that all plants in a box were exposed to the same water level irrespective of plant size, leaf area and stomatal conductance (Verslues et al., 2006).

The initial weight of seedlings was recorded before transplanting into soil. We first compared the growth under well-watered conditions (85% of field capacity) for 5 weeks in the growth chamber at 16 h photoperiod. The temperature varied between 28 and 33°C, while relative air humidity ranged between 47 and 71%. Boxes were weighed every day and the amount of water lost by evapotranspiration was added to maintain 85% of field capacity. Plant height was measured weekly from the soil surface to the apical bud, and the numbers of leaves and branches were counted after three weeks. Leaf length, leaflet length and width were measured after 5 weeks of growth under well-watered conditions. Likewise, stomatal size and density were determined on 50 plants (diploid and polyploid) and two randomly chosen leaflets per seedling. Leaflets were stained with Toluidine Blue O dissolved in 0.05% of benzoate buffer and water at pH 4.4 (O’Brien and McCully, 1981) and viewed with a microscope (Olympus Cx40 RF 200, Japan) under ×40 magnification. Two microscopic grids were examined per leaflet, totaling 200 counts. To determine the size of stomata, 20 random individual stomata per seedling were measured (scale bar 100 μm) at magnification ×40 and the mean stomatal length and density was calculated.

After 5 weeks, drought stress was applied by reducing the amount of added water to 47% of field capacity. In a pilot study, this was shown to be close to the wilting point. Again, in order to maintain the field capacity at 47%, boxes were weighed every day and the amount of water lost was added. Plant height was recorded weekly for 6 weeks and the fresh and dry biomasses of seedlings (after drying at 80°C for 48 h) were assessed at the end of the experiment.

### Ploidy Level Assessment

For 59 trees from the Dahra field trial, twigs of 20 cm length with vegetative buds were collected in April 2014 and placed in a lab with their proximal end in water. After 2 weeks of forcing, one complete leaf was collected from each twig and analyzed by flow cytometry. Seeds descending from the mother trees in the Dahra field trial were germinated as described above, and flow cytometry was performed on 3 weeks old leaves in the lab.

Flow cytometry was performed using a Partec PA II flow cytometer equipped with an HBO-100 mercury arc lamp (Partec GmbH, Germany) and filter combination for DAPI staining (Partec 06-03-310). Fresh leaf samples of two-weeks-old plants or from forced twigs were chopped for 30 s in a petri dish containing 0.6 ml of Citric acid buffer and left for 5 min to allow nuclei release. The nuclei were stained by adding 2.5 ml of fluorescent solution containing 5 μM DAPI (4.6-diamino-2-phenylindol dihydrochlorid) and left for another 5 min (Otto, 1990). The suspension of nuclei was passed through a nylon filter with pore size 50 μm to remove large debris. The DAPI binds to the A–T bases of DNA and the intensity of the fluorescence emitted will reflect the number of bounds and, therefore, also the DNA content in such DAPI-labeled nuclei. The relative fluorescence of total DNA of single nuclei was analyzed and in each sample the DNA content of 5000 nuclei was checked. Samples of *Miscanthus sinensis* with known ploidy (diploid) was used as an internal standard. The standard produced two peaks: a major peak corresponding to 2× DNA quantities of the majority of the cells and a minor peak corresponding to 4× DNA quantities from cells in mitotic interphase.

The gain was adjusted so that the peak of diploid *A. senegal* was localized on channel 50 corresponding to one large (2C) and one small (4C) peak. Plants were regarded as tetraploids if histograms showed one major 4C peak, a small 8C peak and no 2C peak.

To assess the exact DNA content of the genome, a subset of 26 leaf samples from 13 DAPI-examined plants (3 diploids, 3 triploids, 3 tetraploids, 1 pentaploid, and 3 hexaploids) was analyzed using flow cytometry with propidium iodide dye according to the protocol by Doležel et al. (2007).

All 634 living trees in the field trial were genotyped with 8 polymorphic microsatellite markers (SSR) as described in Diallo et al. (2015). By comparing the flow cytometry results with the SSR markers for the 59 mature trees, we concluded that polyploids could always be separated from diploids by the presence of more than two alleles per locus in at least 1 of the 8 loci (Appendix 1). Based on their SSR genotypes, we therefore assigned all 634 trees in the field trial to either diploid or polyploid status, but not distinguishing between tri-, tetra-, penta-, or hexaploids.

### Statistics

For the data from the field trial, a generalized linear analysis of variance was applied to test differences between the growth of diploid and polyploid trees in the trial. Trees that were assessed in 1995 but were not alive in 2012 were excluded, as their ploidy level was unknown. The analysis included the effects of ploidy level (diploid or polyploid), site of origin (Daiba, Diamenar, Kidira, or Ngane) and block (30 levels) according to the following model:

Y=Ploidy+Site⁢ of⁢ origin+Block+Error

where the effects of ploidy and site of origin were considered as fixed, block was considered as random, and the error followed a normal distribution with expectation zero. The average performance of diploids and polyploids was estimated as least square means from the analysis, i.e., averages corrected for systematic differences among provenances.

Data from the growth chamber experiment were analyzed in two steps. The first step was a model with the effects of descendance and blocks:

Y=Descendance+Block+Error

where descendance was considered a fixed effect and the block was considered as random. From this model, we calculated the least square mean values of all variables for each descendance. Due to the limited number of samples for stomatal density and length, calculation of least square means was not possible and instead the mean values were calculated. Mean values were also calculated for seed traits. Next, as each descendance was either diploid or polyploid and originated from one of the four sites of origin, we applied the following model:

Y=Ploidy+Site⁢ of⁢ origin+Error

where *Y* denotes the least square means (or means) of the descendant families from model (2), and ploidy and site of origin were considered as fixed effects.

All analyses were performed using the GLM procedure in the SAS 9.3 Software (Sas Institute Inc, 2014). Assumptions of variance homogeneity and normality of residuals were accepted for all the studied characters based on visual inspection of residual plots. However, for the ratio between fresh and dry weight in the growth chamber experiment a single outlier (Kidira family 7) was identified and deleted from the data.

## Results

### Ploidy Level

Both diploid and polyploid individuals were found among trees from all four origins, but at very different frequencies: 136 of 164 trees (83%) originating from Ngane were polyploid, compared to 3 of 178 trees in Diamenar (2%), 14 of 146 trees in Daiba (10%) and 16 of 117 trees in Kidira (14%).

Flow cytometric analyses of seedlings allowed separation between diploid, triploid, tetraploid, pentaploid, and hexaploid individuals corresponding to a mean 2C DNA content of 1.25 ± 0.02, 1.96 ± 0.05, 2.60 ± 0.03, 3.19, and 3.83 ± 0.08 pg, respectively. All offspring from diploid mothers were diploid, while tetraploid mothers produced either offspring with the same ploidy level (tetraploid) or higher levels (pentaploid, hexaploid, and octaploid). Seeds from the same pod collected on the tetraploid mother NG16_B19 gave rise to one tetraploid and one hexaploid seedling, while all seedlings coming from the tetraploid mother DA15_B3 were hexaploid. Of 13 offspring tested from the tetraploid parent NG10_B8, 11 were tetraploid, one was pentaploid, and one was hexaploid; of the 16 plants examined from the tetraploid mother NG20_B3, one was octaploid, and 15 were tetraploid. Out of three seedlings tested from the triploid individual DA1_17, two were triploid and one was tetraploid (Appendix 1).

### Growth Differences Between Diploid and Polyploid Trees in the Progeny Trial

**FIGURE 2:**
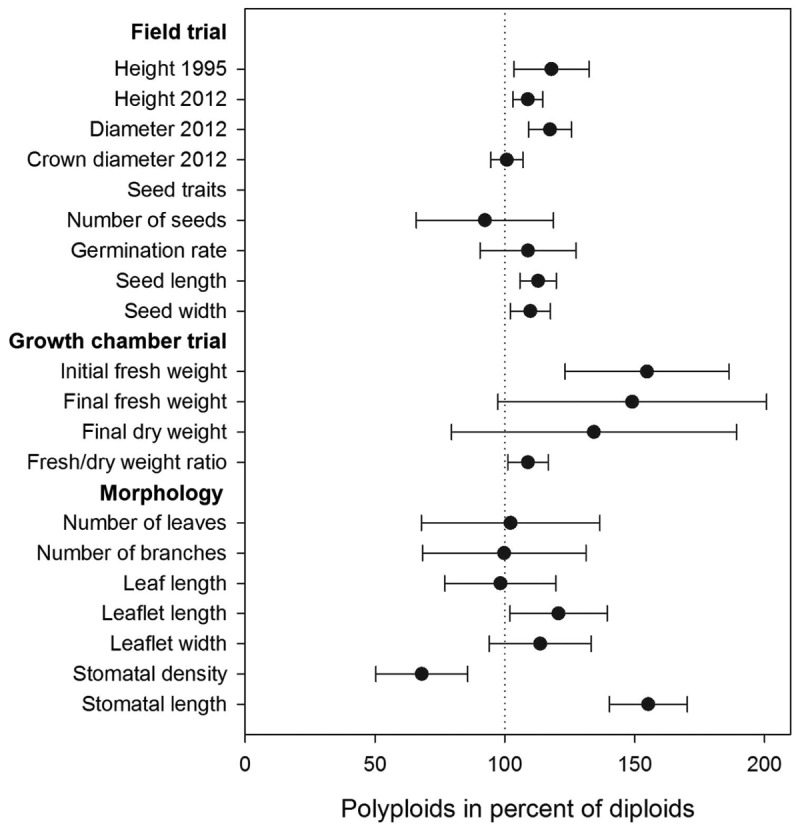
**Performance of polyploids expressed in percent of diploid performance.** The vertical dotted line (100%) denotes diploids. Error bars denote the 95% confidence limits of differences between polyploids and diploids.

The polyploid trees in the Dahra trial were significantly taller than the diploid trees 1 year after planting in 1995. In 2012, after 18 years, differences between diploid and polyploid trees for both height and trunk diameter were still significant, whereas crown diameter did not differ between ploidy levels. The superiority of polyploids compared to diploids based on least square mean estimates after 1 year was 18% for height, while 9% for height, and 17% for trunk diameter after 18 years. The true differences between average of diploid and polyploid trees (i.e., not corrected for systematic effects of provenances) were substantially larger: 49% for height at age 1; and 15 and 24% for height and diameter, respectively, at age 18 (**Table 1**; **Figure 2**).

**Table 1:** *F*-tests for significance in growth traits between ploidy levels and provenances in the field trial and LS estimated averages of diploid and polyploid trees.

	Ploidy level			Provenance			Ploidy (average performance)			
			
Traits	Df	*F*	*P > F*	Df	*F*	*P > F*	Diploid (LS mean)	Polyploid (LS mean)	Diploid (Mean)	Polyploid (Mean)
Height 1995 (cm)	1; 29	5.96	0.015	3; 29	14.61	0.001	38.2 (*1.18*)	45.0 (*2.29*)	38.2 (*16.3*)	57.0 (*22.3*)
Height 2012 (m)	1; 29	9.52	0.002	3; 29	4.24	0.006	4.52 (*0.05*)	4.92 (*0.11*)	4.57 (*0.05*)	5.25 (*0.07*)
Diameter 2012 (cm)	1; 29	17.29	0.001	3; 29	3.64	0.013	11.86 (*0.19*)	13.92 (*0.41*)	11.57 (*0.16*)	14.34 (*0.28*)
Crown diameter 2012 (m)	1; 29	0.06	0.804	3; 29	1.76	0.152	5.50 (*0.07*)	5.55 (*0.14*)	5.40 (*0.06*)	5.61 (*0.09*)


*Least square means (LS means), simple average (Mean), and standard errors (SE) are estimated from the analysis of variance. Df, Degrees of freedom; *F*, *F*-value. Italics are meant to denote standard errors (SE).*

### Phenotypic Differences between Diploid and Polyploid Seedlings in the Drought Stress Test

Polyploids differed significantly from diploids in seed length and width, initial fresh weight (at week 0 when transplanted to the boxes), leaflet length, stomatal density, and length. Heights were similar until weeks 10 and 11. Total fresh weight was borderline significant at the end of the trial, and the fresh weight/dry weight ratio differed significantly between ploidy levels (**Table 2**; **Figure 2**).

**Table 2:** *F*-tests for significance of morphological differences between ploidy levels and provenances of *Acacia senegal* in the growth chamber trial.

Traits	Ploidy level			Ploidy LS means	
		
	Df; error	*F*	*P* > *F*	Diploid	Polyploid
**Seed traits**					
Number of seeds per pod	1; 55	0.34	0.56	4.4 (*0.2*)	4.1 (*0.5*)
Seed length (mm)	1; 49	13.6	0.0006	7.7 (*0.1*)	8.7 (*0.2*)
Seed width (mm)	1; 49	6.5	0.01	7.8 (*0.1*)	8.6 (*0.2*)
Germination rate (%)	1; 55	0.94	0.34	89 (*3*)	95 (*6*)
**Growth and morphology at 87% field capacity**					
Initial total fresh weight (g)	1; 49	12.2	0.001	0.17 (*0.01*)	0.27 (*0.02*)
No. leaves	1; 48	0.02	0.89	16.0 (*1.0*)	16.3 (*2.2*)
No. branches	1; 48	0.00	0.99	6.2 (*0.4*)	6.2 (*0.8*)
Leaf length (cm)	1; 31	0.03	0.87	1.9 (*0.1*)	1.9 (*0.2*)
Leaflet length (mm)	1; 31	5.1	0.03	6.1 (*0.2*)	7.3 (*0.5*)
Leaflet width (mm)	1; 31	2.0	0.17	1.8 (*0.1*)	2.1 (*0.1*)
Stomatal density (mm^-2^)	1; 31	13.7	0.0008	204 (*7*)	139 (*15*)
Stomatal lenght (μm)	1; 31	57	<0.0001	46 (*1*)	71 (*3*)
**Growth at 47% field capacity**					
Height at week 8 (cm)	1; 48	0.97	0.33	15.9 (*0.6*)	17.6 (*1.4*)
Height at week 9 (cm)	1; 48	2.4	0.13	17.2 (*0.7*)	20.0 (*1.5*)
Height at week 10 (cm)	1; 48	4.1	0.05	18.1 (*0.7*)	22.2 (*1.6*)
Height at week 11 (cm)	1; 48	6.3	0.02	19.1 (*0.8*)	24.5 (*1.7*)
Total fresh weight (g)	1; 48	3.6	0.06	1.0 (*0.1*)	1.6 (*0.2*)
Total dry weight (g)	1; 48	1.6	0.21	0.33 (*0.03*)	0.44 (*0.07*)
Fresh weight/dry weight ratio	1; 47	5.4	0.03	3.30 (*0.05*)	3.60 (*0.10*)


*Least square means (LS means) and standard errors (SE) are given for the two ploidy levels. Df, Degrees of freedom; *F*, *F*-value. Italics are meant to denote standard errors (SE).*

The morphological parameters showed that the polyploids tended to be larger than the diploids (**Table 2**). Seed length and width were 12 and 10% larger in polyploids compared to diploids, respectively. Polyploids had leaflets that on average were 19% longer than in diploid individuals, and the differences in stomatal density and length were pronounced: polyploid individuals were characterized by 54% wider stomata but with lower stomatal density (31% less) compared to diploids.

**FIGURE 3:**
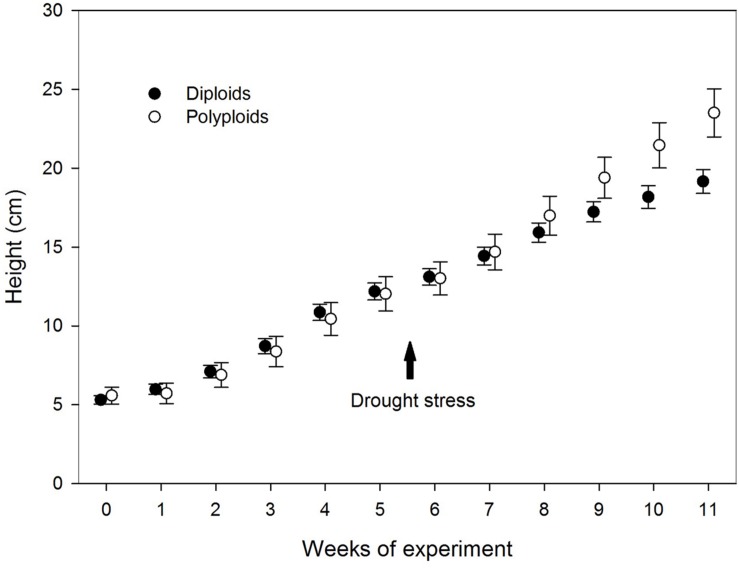
**Variation in height between diploid (filled circles) and polyploid seedlings (open circles) of *A. senegal* under drought stress conditions.** The arrow indicates onset of the drought stress, while error bars denote SD.

Prior to drought stress, the plant height was similar for both ploidy levels, but after 10 and 11 weeks (corresponding to 5 and 6 weeks of water deficit), the tetraploids had grown taller than diploids (22 and 28%, respectively) (**Table 2**; **Figure 3**). At the end of the trial, polyploid *A. senegal* seedlings had 60% larger fresh weight than diploids. Differences in dry weight were smaller because the fresh weight/dry weight ratio was 9% larger in polyploids than in diploids.

## Discussion

### Evolution of Polyploidy in *A. senegal*

Our results showed that *A. senegal* can occur in more than two levels of ploidy, which supplement the results of Assoumane et al. (2013) and Odee et al. (2015) who reported diploid and tetraploid individuals. In our study, we found that diploids were most frequent followed by tetraploids. Pentaploids, triploids, and hexaploids were also present among seedlings although in small quantities.

Based on small sample sizes, Odee et al. (2015) showed co-existence of ploidy types. This result is qualified by the present study, where we show that the frequency of polyploid individuals can vary significantly among natural populations in Senegal. Polyploids were dominant in the population from the central Senegal (Ngane) characterized by saline soils, whereas higher proportions of diploids were found in Diamenar (North), Daiba (North-east), and Kidira (South-east). Nevertheless, a few diploid individuals from the saline site (Ngane) were also identified and polyploids occurred in low frequency in non-saline areas (Kidira, Diamenar, and Daiba sites).

Our data comparing the ploidy level of trees and their offspring indicated only very limited hybridization between cytotypes, even in a setup where diploid and polyploid trees were grown side by side in a field trial. Further, offspring from diploid mothers were always diploid suggesting that hybridization between cytotypes with diploid maternal trees must be very rare if at all possible. The higher ploidy levels occasionally found in offspring from tetraploid mothers on the other hand suggest that tetraploid mothers rather frequently produce some gametes that are unreduced. Hexaploid seedlings from tetraploid mothers may have been formed by unreduced egg cells (4*n*) sired by reduced pollen gametes from a tetraploid pollen donor. The identified pentaploid seedling could have been formed by a pollination event involving a hexaploid pollen donor from the field trial (however not among the trees that were tested with FCM). Alternatively the pentaploid seedling could originate from a fusion between an unreduced egg cell from the tetraploid mother and a reduced pollen gamete from a diploid pollen donor i.e., reflecting cytotype hybridiziation. In relation to this aspect, we found a single triploid mother DA1_B17 which might have been formed by cytotype hybridization in the previous generation. This triploid tree produced both triploid and tetraploid seedlings. Triploids have previously been reported when conspecific diploids and tetraploids co-occur in the same area as, e.g., *Chameron angustifolum* (Husband, 2004) and may play an important role as a triploid-bridge allowing gene flow through mating between diploid and polyploid individuals (Henry et al., 2005) with recurrent polyploid formation in the population.

The frequency of triploid seedlings (2 out of 162 – both from a triploid mother) observed in our study is, however, low compared to the reported frequencies of triploids in species with mixed populations (range of 2–22%) as reviewed by Soltis et al. (2010). Also, no triploid seedlings were observed from either diploid or tetraploid mother trees, which support the presence of a significant reproductive barrier. Stebbins (1971) predicted that mating between ploidy levels is likely only from diploid fathers to polyploid mothers. Unidirectional mating from diploids to tetraploids is known from other species complexes such as *Sorghum* (diploid *Sorghum bicolor* and tetraploid *S. halepense*) (Arriola and Ellstrand, 1996), *Capsella rubella* (to the allotetraploid descendant *C. bursa-pastoris*) (Slotte et al., 2008), and *Arabidopsis arenosa* (Jørgensen et al., 2011). As pollen is aggregated in polyades in *A. senegal* and the stigmatic cavity is cup-shaped (Tandon et al., 2001) morphological size differences between cytotypes could also restrict hybridization. Additional detailed studies are needed to clarify the strength of the reproductive barriers between cytotypes of *A. senegal*, and if pollination is always unidirectional under natural conditions.

### Adaptive Potential and Evolutionary Success of Polyploids

Polyploid trees often occupy drier habitats than their diploid relatives (e.g., Li et al., 1996; Pettigrew et al., 2012). Experiments have shown different performance of diploids and polyploids under drought stress, but are limited by their short duration and experimental setup (Li et al., 1996, 2009). Unfortunately, there is very limited evidence from long-term field trials on the relative performance of trees with different ploidy levels. The faster growth of polyploid *A. senegal* in our study is to our knowledge the first observation of superiority of mature natural polyploid trees in a field trial and indicates that at least under some conditions, trees with high ploidy levels will have an adaptive advantage. Although the effects of ploidy level and origin were to some extent confounded due to the observed unequal distribution of polyploids, our statistical analysis showed that the positive effect of being polyploid remained even when accounting for the effect of origin.

The field trial represents relatively dry conditions close to the Northern limit of distribution of the species toward the Sahara desert. Since the relative performance of diploid and polyploid *A. senegal* has not been investigated under wetter conditions, it is not possible to conclude whether the better growth is found only under dry conditions or indicates a general superior performance.

Still, the growth chamber experiment showed that polyploid plants only grew faster than diploids after the plants were subjected to water stress. The limited number of polyploid plants did not allow us to include a control treatment where seedlings were continuously raised without water stress, and we hence do not separate potential ontogenetic effects from effects of drought. The question of whether the adaptive advantage of polyploids is limited to dry conditions or applies over a broader range of environments therefore remains unresolved. Reciprocal experiments (‘optimal’ versus a single abiotic stress factor) to test the relative performance of di- and polyploids under different stress situations are needed to conclude whether polyploids are generally superior or if it is only the case under dry conditions (cf. Soltis et al., 2010). Investigations in other heteroploid tree species or species complexes are needed to reveal if the observed effects of polyploid in *A. senegal* reflect a so-far undiscovered pattern in trees species growing under stressful conditions in Sahel.

Polyploidy is often associated with a difference in plant phenotype (e.g., increase in cell size, enlarged floral structure, pollen, stomata, and robust stems) when compared to the diploid relatives (Ramsey and Schemske, 1998; Madlung, 2013). In our study, we found the first evidence of phenotype differentiation between cytotypes in *A. senegal*, as seeds, leaflets and stomata were larger and stomatal densitites were smaller in tetraploids of *A. senegal*. This confirms results reported for other tree species, such as *A. digitata* (Pettigrew et al., 2012), *B. papyrifera* (Li et al., 1996)*, A. mangium* (Harbard et al., 2012) and *A. maernsii* (Beck et al., 2003). Studies based on neopolyploids have shown that many of these polyploid characteristics are directly linked to increased genome size (e.g., Harbard et al., 2012). It is unknown whether the size differences in *A. senegal* are caused by increased genome size, increased genetic diversity or a combination of both.

The observed phenotypic differences are likely to lead to differences in physiology, as gas movement in and out of leaves is affected by leaf size, size, and distribution of stomata. It has been hypothesized that the fewer, but larger stomata observed in polyploids can change stomatal conductance and confer increased water use efficiency to polyploids under drought stress (e.g., Li et al., 1996, 2009; Pettigrew et al., 2012). Assuming that width and depth of the stomatal pore is proportional to the length, it can easily be estimated following Franks and Farquhar (2007) and Franks and Beerling (2009) that stomatal conductance is expected to be 5% larger in polyploids than diploids. On the other hand, estimates based on leaf dimensions (Nobel, 2009) suggest that leaf boundary layer conductance will be reduced by 9% in polyploids compared to diploids. Hence, the effects of leaf size tend to negate effects of changed stomatal size and density, and the expected overall effects of ploidy level on water use efficiency are therefore unclear. Detailed anatomical studies, coupled with assessments of gas exchange on trees with different levels of ploidy will be important in order to infer on potential mechanisms behind the putative selective advantage of polyploidy in *A. senegal*. For example, in the herbaceous perennial *C. angustifolium*, Maherali et al. (2009) found that tetraploids characterized by large stomata and wide xylem vessels did not differ from diploids in stomatal conductance and gas exchange when grown under drought conditions. However, increased hydraulic conductivity was believed to cause increased drought resistance of tetraploids in this species.

Another observed phenotypic difference with potential physiological consequences is the larger fresh to dry weight ratios of polyploids compared to diploids (**Table 2**) causing the ploidy levels to differ almost significantly in fresh weight, but not in dry weight. High water contents indicate either a larger capacity for osmotic adjustment or an increased elasticity of cell walls (Verslues et al., 2006) and Li et al. (1996) suggested that polyploids might have a larger ability to adjust their osmotic potential under drought stress. If this is indeed the case, it may explain part of the better performance of polyploids in *A. senegal*.

## Conclusion

The co-existence of different ploidy levels in natural populations was confirmed while the pattern of segregation supports that gene flow between cytotypes is limited. Our results document increased growth of polyploid *A. senegal* both in the field trial and under growth chamber conditions, but it remains to be verified if superiority of polyploids is expressed only under relatively dry conditions or applies more generally.

## Author Contributions

AD, LN, EK, and AR conceived the ideas; AD, LN, and KP collected the data; AD, EK, and AR carried out the statistical analyses; all authors analyzed and interpreted the data, and all authors contributed to writing of the paper.

## Conflict of Interest Statement

The authors declare that the research was conducted in the absence of any commercial or financial relationships that could be construed as a potential conflict of interest.
