# Supplementary material for: Polyploidy can Confer Superiority to West African Acacia senegal (L.) Willd. Trees
Source: Front Plant Sci. 2016 Jun 14;7:821. doi: 10.3389/fpls.2016.00821 (PMC4906048; doi:10.3389/fpls.2016.00821)
Supplement: Supplementary file 1 [file Data_Sheet_1.DOCX]

**Appendix 1.** Ploidy levels in a sub-set (76 parents) of *Acacia senegal* in the progeny trial and in their offspring revealed by eight polymorphic microsatellites and flow cytometry (FCM) respectively

| Parent ploidy levels | | | | | |  | Offspring ploidy levels | | | | | |  |
| --- | --- | --- | --- | --- | --- | --- | --- | --- | --- | --- | --- | --- | --- |
| Provenance | Family | Ind. | No. loci with 1-2 alleles | No. loci with more than 2 alleles | FCM on twig | No. Pods tested | No. offspring tested | Diploid | Triploid | Tetra | Penta | Hexa | Octo |
|  |  |  |  |  |  |  |  | (2n) | (3n) | (4n) | (5n) | (6n) | (8n) |
| Ngane | NG4 | *B14 | 5 | 3 | Tetraploid | 1 | 2 | - | - | 2 | - | - | - |
| Ngane | NG4 | B16 | 4 | 4 | Na | 5 | 11 | - | - | 10 | - | 1 | - |
| **Ngane** | **NG7** | ***B22** | **8** | **0** | **Diploid** | **1** | **2** | **2** | - | **-** | - | - | - |
| Ngane | NG10 | *B8 | 7 | 1 | Tetraploid | 6 | 13 | - | - | 11 | 1 | 1 | - |
| **Ngane** | **NG11** | ***B17** | **8** | **0** | **Diploid** | **1** | **2** | **2** | - | **-** | - | - | - |
| Ngane | NG14 | B1 | 5 | 3 | Na | 5 | 12 | - | - | 11 | - | 1 | - |
| Ngane | NG14 | *B2 | 5 | 3 | Tetraploid | 1 | 2 | - | - | 2 | - | - | - |
| Ngane | NG15 | *B16 | 5 | 3 | Tetraploid | 1 | 2 | - | - | 2 | - | - | - |
| Ngane | NG16 | B3 | 5 | 3 | Na | 4 | 9 | - | - | 9 | - | - | - |
| Ngane | NG16 | *B19 | 4 | 4 | Tetraploid | 1 | 2 | - | - | 1 | - | 1 | - |
| Ngane | NG17 | *B8 | 5 | 3 | Tetraploid | 1 | 2 | - | - | 2 | - | - | - |
| Ngane | NG18 | *B25 | 4 | 4 | Tetraploid | 1 | 2 | - | - | 2 | - | - | - |
| Ngane | NG19 | B1 | 4 | 4 | Na | 4 | 8 | - | - | 6 | - | 2 | - |
| Ngane | NG19 | * B24 | 4 | 4 | Tetraploid | 1 | 2 | - | - | 2 | - | - | - |
| Ngane | NG20 | *B3 | 4 | 4 | Tetraploid | 6 | 16 | - | - | 15 | - | - | 1 |
| Ngane | NG21 | * B4 | 4 | 4 | Tetraploid | 6 | 13 | - | - | 12 | - | 1 | - |
| Ngane | NG22 | *B6 | 5 | 3 | Tetraploid | 1 | 2 | - | - | 2 | - | - | - |
| Ngane | NG25 | *B30 | 4 | 0 | Diploid | 4 | 10 | 10 | - | - | - | - | - |
| Ngane | NG26 | *B17 | 4 | 4 | Tetraploid | 1 | 2 | - | - | 2 | - | - | - |
| Diamenar | DIA2 | B1 | 8 | 0 | Na | 1 | 6 | 6 | - | - | - | - | - |
| Diamenar | DIA2 | *B2 | 8 | 0 | Diploid | 1 | 2 | 2 | - | - | - | - | - |
| Diamenar | DIA6 | *B2 | 8 | 0 | Diploid | 1 | 2 | 2 | - | - | - | - | - |
| Diamenar | DIA6 | B3 | 8 | 0 | Na | 1 | 4 | 4 | - | - | - | - | - |
| Diamenar | DIA7 | *B27 | 8 | 0 | Na | 1 | 2 | 2 | - | - | - | - | - |
| Diamenar | DIA8 | *B21 | 8 | 0 | Na | 1 | 2 | 2 | - | - | - | - | - |
| Diamenar | DIA11 | * B5 | 8 | 0 | Na | 1 | 2 | 2 | - | - | - | - | - |
| Diamenar | DIA13 | *B26 | 8 | 0 | Na | 1 | 2 | 2 | - | - | - | - | - |
| Diamenar | DIA14 | *B6 | 8 | 0 | Na | 1 | 2 | 2 | - | - | - | - | - |
| Diamenar | DIA15 | B8 | 8 | 0 | Na | 1 | 2 | 2 | - | - | - |  | - |
| Diamenar | DIA15 | *B21 | 8 | 0 | Diploid | 1 | 2 | 2 | - | - | - | - | - |
| Diamenar | DIA17 | *B17 | 8 | 0 | Na | 1 | 2 | 2 | - | - | - | - | - |
| Diamenar | DIA18 | B4 | 8 | 0 | Diploid | 1 | 2 | 2 | - | - | - | - | - |
| Diamenar | DIA20 | *B23 | 8 | 0 | Na | 1 | 2 | 2 | - | - | - | - | - |
| Diamenar | DIA22 | *B6 | 8 | 0 | Na | 1 | 3 | 3 | - | - | - |  | - |
| Diamenar | DIA22 | *B14 | 8 | 0 | Na | 1 | 2 | 2 | - | - | - | - | - |
| Diamenar | DIA26 | *B2 | 8 | 0 | Na | 1 | 2 | 2 | - | - | - | - | - |
| Diamenar | DIA27 | *B5 | 8 | 0 | Na | 1 | 2 | 2 | - | - | - | - | - |
| Diamenar | DIA29 | *B5 | 8 | 0 | Na | 1 | 2 | 2 | - | - | - | - | - |
| Daiba | DA1 | B17 | 7 | 1 | Triploid | 1 | 3 | - | 2 | 1 | - | - | - |
| Daiba | DA1 | *B19 | 8 | 0 | Diploid | 1 | 5 | 5 | - | - | - | - | - |
| Daiba | DA2 | *B5 | 8 | 0 | Diploid | 1 | 3 | 3 | - | - | - | - | - |
| Daiba | DA4 | *B12 | 8 | 0 | Diploid | 1 | 3 | 3 | - | - | - | - | - |
| Daiba | DA4 | *B22 | 7 | 1 | Na | 1 | 3 | - | - | 3 | - | - | - |
| Daiba | DA6 | *B6 | 8 | 0 | Diploid | 1 | 2 | 2 | - | - | - | - | - |
| Daiba | DA7 | *B1 | 8 | 0 | Diploid | 1 | 2 | 2 | - | - | - | - | - |
| Daiba | DA8 | *B17 | 8 | 0 | Diploid | 1 | 2 | 2 | - | - | - | - | - |
| Daiba | DA13 | *B9 | 8 | 0 | Diploid | 1 | 2 | 2 | - | - | - | - | - |
| Daiba | DA15 | *B3 | 7 | 1 | Tetraploid | 1 | 2 | - | - | - | - | 2 | - |
| Daiba | DA15 | B11 | 8 | 0 | Diploid | 1 | 5 | 5 | - | - | - | - | - |
| Daiba | DA16 | *B15 | 8 | 0 | Diploid | 1 | 2 | 2 | - | - | - | - | - |
| Daiba | DA17 | *B2 | 8 | 0 | Diploid | 1 | 2 | 2 | - | - | - | - | - |
| Daiba | DA18 | B2 | 8 | 0 | Diploid | 1 | 4 | 4 | - | - | - | - | - |
| Daiba | DA18 | B5 | 8 | 0 | Diploid | 1 | 1 | 1 | - | - | - | - | - |
| Daiba | DA19 | *B6 | 8 | 0 | Diploid | 1 | 2 | 2 | - | - | - | - | - |
| Daiba | DA20 | *B13 | 8 | 0 | Diploid | 1 | 4 | 4 | - | - | - | - | - |
| Daiba | DA25 | *B8 | 8 | 0 | Diploid | 1 | 3 | 3 | - | - | - | - | - |
| Daiba | DA25 | B12 | 8 | 0 | Diploid | 1 | 3 | 3 | - | - | - | - | - |
| Daiba | DA26 | B2 | 8 | 0 | Diploid | 1 | 5 | 5 | - | - | - | - | - |
| Daiba | DA26 | B9 | 8 | 0 | Diploid | 1 | 3 | 3 | - | - | - | - | - |
| Kidira | K1 | *B3 | 8 | 0 | Diploid | 1 | 2 | 2 | - | - | - | - | - |
| Kidira | K3 | *B2 | 8 | 0 | Diploid | 1 | 2 | 2 | - | - | - | - | - |
| Kidira | K4 | *B23 | 8 | 0 | Diploid | 1 | 2 | 2 | - | - | - | - | - |
| Kidira | K5 | *B7 | 8 | 0 | Diploid | 1 | 2 | 2 | - | - | - | - | - |
| Kidira | K7 | *B8 | 8 | 0 | Diploid | 1 | 2 | 2 | - | - | - | - | - |
| Kidira | K8 | *B7 | 8 | 0 | Diploid | 1 | 2 | 2 | - | - | - | - | - |
| Kidira | K9 | *B5 | 8 | 0 | Diploid | 1 | 2 | 2 | - | - | - | - | - |
| Kidira | K14 | *B6 | 8 | 0 | Diploid | 1 | 2 | 2 | - | - | - | - | - |
| Kidira | K16 | *B15 | 8 | 0 | Diploid | 1 | 4 | 4 | - | - | - | - | - |
| Kidira | K17 | B14 | 8 | 0 | Diploid | 1 | 2 | 2 | - | - | - | - | - |
| Kidira | K20 | *B9 | 8 | 0 | Diploid | 1 | 4 | 4 | - | - | - | - | - |
| Kidira | K21 | B13 | 8 | 0 | Diploid | 1 | 5 | 5 | - | - | - | - | - |
| Kidira | K21 | *B22 | 4 | 4 | Tetraploid | 1 | 2 | - | - | 2 | - | - | - |
| Kidira | K22 | *B9 | 8 | 0 | Diploid | 1 | 2 | 2 | - | - | - | - | - |
| Kidira | K23 | B3 | 6 | 2 | Na | 1 | Na | Na | - | Na | - | - | - |
| Kidira | K23 | B6 | 8 | 0 | Diploid | 1 | 4 | 4 | - | - | - | - | - |
| Kidira | K25 | *B16 | 4 | 4 | Tetraploid | 1 | 2 | - | - | 2 | - | - | - |

In bold: The pure diploid Ngane families (NG7 & NG11). Dark grey: triploid mother producing both triploid and tetraploid offspring. Light grey: Families with mixed ploidy levels. Individuals marked with * were used in the drought stress trial. Na: Not assessed.
